# Severe Acute Respiratory Syndrome Coronavirus 2 (SARS-CoV-2) Positive Newborns of COVID-19 Mothers After Dyad-Care: A Case Series

**DOI:** 10.7759/cureus.12528

**Published:** 2021-01-06

**Authors:** Uday P Patil, Parvathy Krishnan, Samira Abudinen-Vasquez, Sheela Maru, Lawrence Noble

**Affiliations:** 1 Pediatrics / Neonatal-Perinatal Medicine, Icahn School of Medicine at Mt. Sinai and New York City Health + Hospitals/Elmhurst, Elmhurst, USA; 2 Pediatrics, Icahn School of Medicine at Mt. Sinai and New York City Health + Hospitals/Elmhurst, Elmhurst, USA; 3 Obstetrics, Gynecology and Reproductive Science, New York City Health + Hospitals/Elmhurst, New York City, USA; 4 Health System Design and Global Health and Obstetrics, Gynecology and Reproductive Science, Icahn School of Medicine at Mount Sinai, New York City, USA

**Keywords:** sars-cov-2, newborns, dyad-care, colocation, covid-19

## Abstract

The novel severe acute respiratory syndrome coronavirus 2 (SARS-CoV-2) infection in newborns is extremely rare, and there is a scarcity of research pertaining to epidemiology, clinical presentation, transmission, and prognosis in this population. We present five newborns who tested positive while colocating with their SARS-CoV-2 positive mothers from March 19 to May 15, 2020, at a large public hospital in Queens, New York that was severely affected by the coronavirus disease 2019 (COVID-19) pandemic. All the newborns subsequently tested negative and remained asymptomatic, including through median outpatient follow-up of three weeks.

## Introduction

During the current coronavirus disease 2019 (COVID-19) pandemic caused by the novel severe acute respiratory syndrome coronavirus 2 (SARS-CoV-2), newborns are at risk of acquiring SARS-CoV-2 infection from their infected mothers [1]. In this case series, we describe five newborns who tested positive for SARS-CoV-2 after birth at NYC Health + Hospitals/ Elmhurst (Elmhurst Hospital), located in Queens, New York City, a Baby-Friendly Hospital that was at the epicenter of the pandemic in the United States during spring and summer of 2020. Due to the unprecedented surge of mothers with a positive test delivering at our hospital after universal maternal testing for SARS-CoV-2 was implemented, we allowed colocation and mother-baby dyad-care, including skin-to-skin care and breastfeeding, for asymptomatic or mildly symptomatic mothers after shared decision making. In-room isolation precautions were followed as per the Centers for Disease Control and Prevention (CDC) and American Academy of Pediatrics (AAP) guidance that included a distance of ≥6 feet between the mother and newborn, placing the newborn in an isolette, use of curtain as a barrier, mother practicing frequent hand hygiene and wearing a face-mask at all times including while breastfeeding [2, 3]. Newborn infants born to these mothers were screened for the virus. Although a limited number of cases in the current literature reveal that most newborns have favorable outcomes, there remain uncertainties concerning the possible transmission of the virus from the mother to the baby [4-6]. This case series of SARS-CoV-2 positive newborns is thus an important contribution to understanding the etiopathology and management of these infants.

## Case presentation

This research was approved under exempt status by the local Institutional Review Board, ethics committee, and the office of research administration. Out of 130 newborns tested at our hospital following the universal testing of their mothers from March 19 to May 15, 2020, five newborns tested positive for SARS-CoV-2. The testing was performed using real-time reverse transcriptase-polymerase chain reaction (RT-PCR) by Bio-Reference assay (Bio-Reference Laboratories Inc, Spring Valley, NY, USA) or the Cepheid Xpert Xpress test (Cepheid, Sunnyvale, CA, USA) in a nasopharyngeal swab. Initially, in the pandemic, newborns born to SARS-CoV-2 positive mothers were tested with one test after their first bath during the hospital stay due to the long turnaround time of the test. As the pandemic continued and the rapid test became available, a second test was performed on newborns at 24 hours of life. 

The demographic and clinical characteristics of these five newborns and their mothers are shown in Tables 1-2. Additional information regarding individual cases is discussed in the supplemental appendix. All five newborns were born to asymptomatic term mothers, with no perinatal complications. Three of the five newborns were born via Cesarean section, and none of them had prolonged rupture of membranes. All the newborns colocated with their mothers who tested positive for SARS-CoV-2 infection during labor and received dyad-care after delivery. The maternal laboratory findings and chest X-rays were unremarkable. The mother of patient number five had D-dimer tested that was within the range for the third trimester of pregnancy. Four newborns tested positive for SARS-CoV-2 soon after birth and were then transferred to an isolation room under the care of the neonatal intensive care unit (NICU) staff for close monitoring with recommended isolation precautions [2, 3]. The mothers were encouraged to pump breastmilk, although none of the newborns in this series received expressed breastmilk. Neonatal laboratory investigations, including a complete blood count (CBC), hepatic function panel (HFP), c-reactive protein (CRP), and procalcitonin (PCT), were obtained (except patient no. 5, who did not get HFP). CBC and HFP remained within the normal range for age. If elevated, CRP and PCT trended down on subsequent testing or were not repeated based on the lack of clinical features suggestive of COVID-19. The same approach was used for the interpretation of chest X-ray findings, which remained unremarkable. These four newborns remained asymptomatic throughout their hospital stay and were discharged after two subsequent PCR tests for SARS-CoV-2 were performed >24 hours apart and resulted negative. Due to the evolving nature of experience around the management of newborns with positive SARS-CoV-2 test and improved turnaround time of the RT-PCR test, the duration of stay in the NICU for these newborns decreased over time. The mothers were encouraged to breastfeed after careful hand hygiene and use of a facemask. Close follow up showed that they continued to remain asymptomatic and well.

**Table 1:** Demographic and clinical characteristics of the mothers of SARS-CoV-2 positive newborns CRP - C-reactive protein; SARS-CoV-2 - severe acute respiratory syndrome coronavirus 2

Maternal demographic and clinical characteristics	Patient 1	Patient 2	Patient 3	Patient 4	Patient 5
Age (years)	30	36	31	22	36
Gravida	5	3	2	3	1
Maternal symptoms					
Fever on admission	No	No	No	No	No
Postpartum fever	No	No	No	No	No
Highest temperature (˚F)	98	98.2	98.9	99.5	98.7
Cough	No	No	No	No	No
Maternal SARS-CoV-2 testing					
Nasopharyngeal swab positive for SARS-CoV-2	Yes	Yes	Yes	Yes	Yes
Gestational age when tested (weeks, days/7)	39 2/7	37 5/7	37 4/7	36 5/7	37 1/7
Maternal laboratory results					
White cell count (x 10^3^/ microliter)	10.84	13.47	13.17	9.86	14.4
Absolute lymphocyte count (x 10^3^/ microliter)	2.88	2.42	1.38	1.92	1.53
Absolute neutrophil count (x 10^3^/ microliter)	7.24	9.17	11.2	7.15	11.8
CRP	n/a	n/a	n/a	n/a	n/a
D-dimer (ng/ml DDU)	n/a	n/a	n/a	n/a	2520
Ferritin	n/a	n/a	n/a	n/a	n/a
Pro-calcitonin	n/a	n/a	n/a	n/a	n/a
Maternal management					
Intensive care unit admission	No	No	No	No	No
Infiltrates on chest x-ray	n/a	n/a	n/a	n/a	n/a
Respiratory support required	No	no	no	No	No
Duration of stay (days)	2	2	2	2	3

**Table 2:** Demographic and clinical characteristics of SARS-CoV-2 positive newborns SARS-CoV-2 - severe acute respiratory syndrome coronavirus 2; NICU - neonatal intensive care unit; COVID-19 - coronavirus disease 2019

Newborn demographic and clinical characteristics	Patient 1	Patient 2	Patient 3	Patient 4	Patient 5
Gender	Male	Female	Male	Male	Female
Gestational age (weeks, days/7)	39 2/7	37 5/7	37 5/7	37 0/7	37 2/7
Birth weight (grams)	3360	3420	3030	3125	2540
APGAR score at 1 min	9	8	9	9	9
APGAR score at 5 min	9	9	9	9	9
Mode of delivery	Vaginal	C-section	Vaginal	C-section	C-section
Instrumentation	No	No	No	No	No
Duration of rupture of membranes (hours)	<1	<1	5	<1	9
Artificial rupture	No	Yes	Yes	Yes	Yes
Premature rupture of membranes	No	No	No	No	No
Resuscitation at birth	No	No	No	No	No
Practices of dyad-care					
Colocated (room-in) with mother	Yes	Yes	Yes	Yes	Yes
Skin-to-skin care	Yes	Yes	Yes	Yes	Yes
Direct breastfeeding	No	Yes	Yes	Yes	Yes
Expressed breast milk	No	No	No	No	No
Supplemental formula	Yes	Yes	Yes	Yes	Yes
Initiation of breastfeeding (hours)	n/a	2	1.5	1.5	1.5
Timing of first bath in hours, hours	4	3	4	2	2
Newborn SARS-CoV-2 testing					
1^st^ SARS-CoV-2 test (hours of life)	1	5	5	3	3
1^st^ SARS-CoV-2 test result	Positive	Positive	Positive	Negative	Positive
2^nd^ SARS-CoV-2 test (hours of life)	26	58	34	24	27
2^nd^ SARS-CoV-2 test result	Negative	Negative	Negative	Positive	Negative
3^rd^ SARS-CoV-2 test (hours of life)	64	83	58	146	48
3^rd^ SARS-CoV-2 test result	Negative	Negative	Negative	Negative	Negative
4^th^ SARS-CoV-2 test (hours of life)	n/a	n/a	n/a	290	n/a
4^th^ SARS-CoV-2 test result	n/a	n/a	n/a	Negative	n/a
Symptoms of COVID-19	No	No	No	n/a	No
Newborn laboratory results					
White cell count (x 10^3^/micro-liter)	13.8	10.7	9.0	n/a	11.3
Absolute lymphocyte count (x 10^3^/ microliter)	2.8	3.5	1.9	n/a	2.5
Absolute neutrophil count (x 10^3^/ microliter)	8.9	5.19	5.69	n/a	7.29
Platelets (x 10^3^/ microliter)	218	209	263	n/a	255
Procalcitonin (ng/mL)	3.37	1.56	1.39	n/a	1.53
C-reactive protein (mg/L)	12.9	8.2	0.4	n/a	0.4
Aspartate aminotransferase (U/L)	71	65	35	n/a	n/a
Alanine aminotransferase (U/L)	24	10	9	n/a	n/a
Management of the newborn					
Respiratory support	No	No	No	No	No
Significant findings on chest x-ray	n/a	No	No	n/a	No
Antibiotics	No	No	No	No	No
Duration of total hospital stay, days	5	4	2	2	2
Duration of NICU stay, days	4	2	1	0	1
Newborn follow-up					
1^st^ in-person follow-up visit (days of life)	8	7	4	4	5
Symptoms on 1^st^ in-person follow up visit	No	No	No	No	No
Breastfeeding at home	No	Yes	Yes	Yes	Yes
1^st^ tele-medicine follow-up (days of life)	12	11	7	8	8
Symptoms on 1^st^ tele-medicine follow up	No	No	No	No	No
2^nd^ tele-medicine follow-up (days of life)	29	25	18	18	20
symptoms of COVID-19 after discharge	No	No	No	No	No

**Figure 1:**
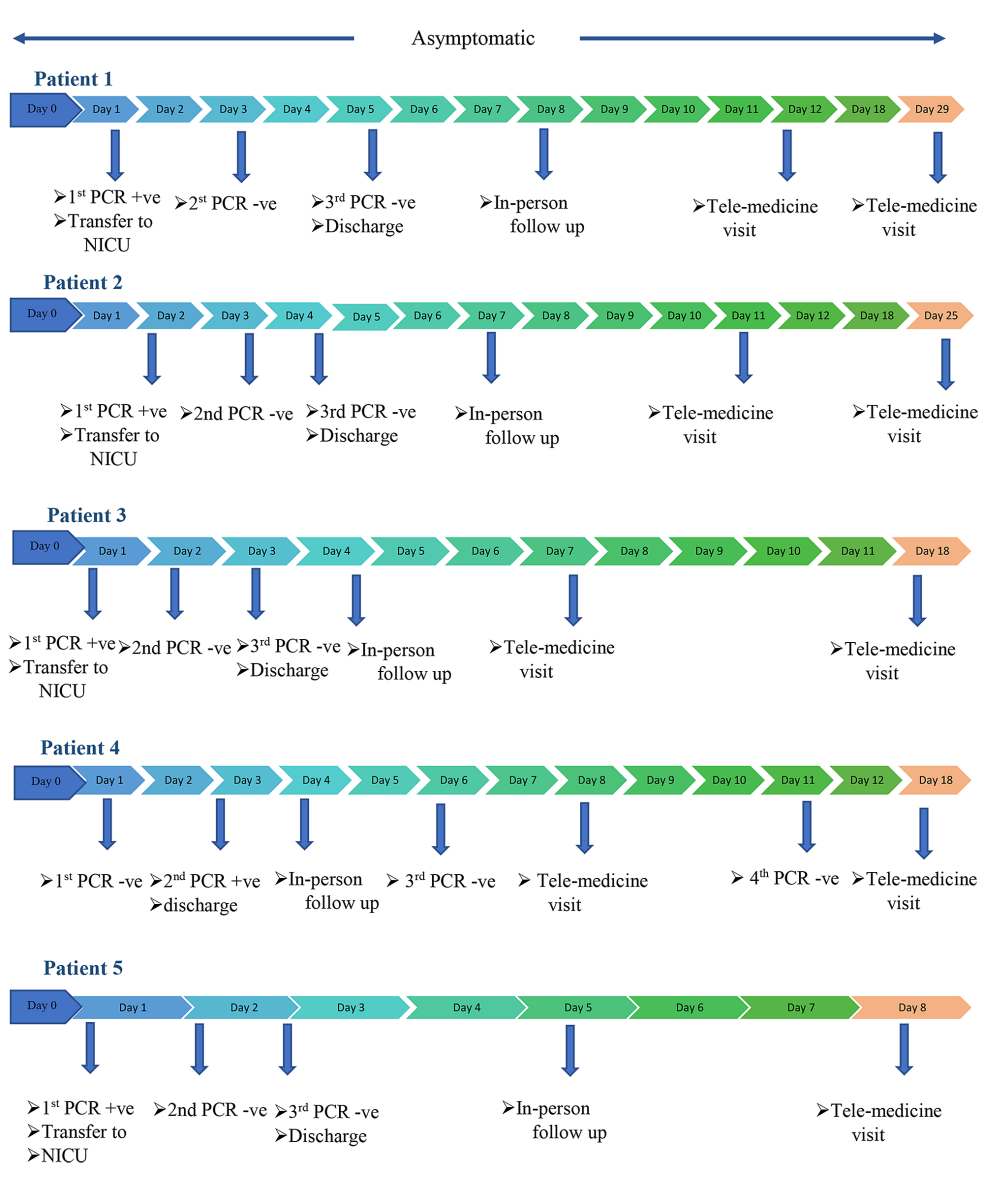
Timeline of testing, management and short-term follow-up of SARS-CoV-2 positive newborns NICU - neonatal intensive care unit; PCR - polymerase chain reaction

One patient (patient no. 4) had an initial negative test; however, the baby’s second PCR, sent after 24 hours of life, was reported as presumptive positive after the newborn was discharged from the hospital. Following this, the newborn was followed in the pediatric infectious diseases (ID) clinic in addition to the general pediatrics clinic. His subsequent oropharyngeal/nasopharyngeal (OP/NP) swab PCR done on days 7 and 12 of life were negative. He remained asymptomatic and well upon follow-up. A timeline of testing, management, and short-term follow-up of the newborns is illustrated in Figure 1.

## Discussion

Our case series describes SARS-CoV-2 detected by PCR in newborns soon after birth following baby-friendly dyad-care practices with their asymptomatic SARS-CoV-2 positive mothers. Although there have been cases of viral nucleic acid detected in newborns after birth, the data regarding infection in newborns after colocation with their SARS-CoV-2 positive mothers are very limited, and none were tested this early after birth [7, 8]. Some experts recommend early testing after birth that may aid in establishing the prevalence and classification of SARS-CoV-2 infection in newborns [9]. Except for one, all newborns in our series received a bath prior to the PCR test, thus decreasing the chances of a positive result due to contamination. Moreover, two newborns in our series were delivered via Cesarian section (C/S) with intact membranes prior to C/S, making the possibility of transmission through the cervicovaginal route unlikely. All five mothers were asymptomatic, and the strict use of in-room isolation precautions for infected mothers, as described above, may have decreased the chances of droplet-based viral transmission. In addition, if the transmission was indeed droplet-based, the subsequent PCR tests should have continued to detect SARS-CoV-2 in these newborns, given the colocation and dyad-care for at least 24 hours preceding the second PCR test. The SARS-CoV-2 PCR resulting as “presumed positive” at 24 hours of life in patient number four could possibly be due to respiratory droplet transmission from the mother. The possibility of environmental contamination cannot entirely be ruled out but seems unlikely given that we followed enhanced-droplet precautions and a strict protocol to dyad-care in SARS-CoV-2 positive mothers, where all staff caring for them used appropriate protective personal equipment (PPE) and changed PPE when switching care between the infected mother and her newborn [3]. In addition, these were the only positive newborn cases among 130 newborns tested at our center during this period.

This raises the possibility of inactive viral particles or even a scant amount of live virus that may have remained in the baby’s nasopharynx or oropharynx after a bath and hence was detected by PCR soon after birth but was not able to replicate or cause infection as evidenced by the subsequent negative PCR results and uneventful clinical course. The reassuring outcomes in all the newborns in our series could be explained based on immature angiotensin-converting enzyme-2 (ACE-2) receptor expression, differences in immune response, and other various mechanisms as proposed by Rawat et al. [10]. We also postulate that the strict in-room isolation precautions, asymptomatic course of the mothers, promoting breastfeeding, and protective antibodies from the infected mother could also have improved newborn outcomes [6]. Recent reports suggest that infants born to SARS-CoV-2 positive mothers may benefit from transplacental immunoglobulin G (IgG) and breastmilk secretory-immunoglobulin A (IgA) antibodies against SARS-CoV-2 [11, 12].

Our series is limited by the lack of antibody titers against SARS-CoV-2 in these newborns who tested positive using PCR test. This could be partially attributed to the lack of widely available and reliable laboratory test for antibodies, especially for immunoglobulin M (IgM) antibodies in newborn infants at the time of our cases. Another limitation of our series is the short follow-up period of these newborns.

## Conclusions

This case series demonstrates that the presence of SARS-CoV-2 nucleic acid may be detected using PCR tests in newborns soon after birth when receiving dyad-care with their asymptomatic SARS-CoV-2 positive mothers. There may be a mother to baby transmission of a scant amount of live virus or inactive viral particles in some cases with possible transplacental route based on the clinical scenarios in our cases and the currently evolving evidence on peripartum transmission. The lack of detection of viral particles in the newborn samples on subsequent PCR tests after dyad-care with their mothers suggests that these asymptomatic mothers may not continue to transmit the SARS-CoV-2 to their newborns via respiratory droplets while colocating if recommended transmission-based precautions are strictly followed. The short-term outcomes of newborns who tested positive for SARS-CoV-2 remain reassuring. Further studies in large cohorts of mother-baby dyads during the COVID-19 pandemic with longer periods of follow-up are necessary to confirm these findings and the significance of early positive tests in newborns.

## Appendices

Summaries of individual cases

Patient 1

A full-term, appropriate for gestational age (AGA) male neonate was born via uncomplicated vaginal delivery to a 29-year-old mother with a history of asthma and mental health problems. The baby was born vigorous and had normal APGAR scores after routine resuscitation at birth. The mother tested positive for SARS-CoV-2 during labor, and she remained asymptomatic throughout her hospital stay. She did not need a chest X-ray, and her laboratory investigations were within normal limits. After skin-to-skin care and colocating with the mother, the newborn received formula feeding at the mother’s request and did not receive breastfeeding or expressed breast milk. His OP/NP swab test resulted positive for SARS-CoV-2 after birth. He was transferred to an isolation room under the Neonatal Intensive Care Unit (NICU) service for close monitoring. A pediatric infectious disease (ID) specialist was consulted. Laboratory investigations, including serial complete blood count (CBC) and hepatic function panel, were reassuring for the age of the baby. After the initial elevation of c-reactive protein (CRP) and procalcitonin (PCT), they trended down the next day. Chest X-ray showed no abnormalities. The newborn remained asymptomatic during the hospital stay and was discharged after two subsequent tests for SARS-CoV-2 performed >24 hours apart resulted negative. Close follow up showed that he continued to remain asymptomatic and well.

Patient 2

An early-term, AGA female neonate, was born via uncomplicated repeat Cesarean section (C/S) to a 36-year-old mother with a history of gestational diabetes mellitus (GDM) controlled with diet. The baby was born vigorous and had normal APGAR scores after routine resuscitation and deep oropharyngeal suctioning at birth. The mother tested positive for SARS-CoV-2 during labor, and she remained asymptomatic throughout her hospital stay. She did not need a chest X-ray, and her laboratory investigations showed mild leukocytosis without lymphopenia. After skin-to-skin care and colocating with the mother, the newborn was breastfed. The newborn was tested following the baby’s first bath using OP/NP swab that detected SARS-CoV-2. The newborn was transferred to an isolation room under the NICU service for close monitoring. A pediatric ID specialist was consulted. Laboratory investigations, including CBC, HFP, and CRP, were reassuring for the age of the baby while PCT was slightly elevated. Chest X-ray showed clear lungs. The newborn remained asymptomatic during the hospital stay and was discharged after two subsequent tests for SARS-CoV-2 performed >24 hours apart resulted negative. She continued to remain asymptomatic and well upon follow-up.

Patient 3

An early-term, AGA female neonate, was born via uncomplicated vaginal delivery to a 31-year-old mother with intrahepatic cholestasis of pregnancy. The baby was born vigorous and had normal APGAR scores after routine resuscitation at birth. The mother tested positive for SARS-CoV-2 during labor, and she remained asymptomatic throughout her hospital stay. Her laboratory investigations were within normal limits. After skin-to-skin care and colocating with the mother, the newborn was breastfed. The newborn was tested following the baby’s first bath using OP/NP swab that detected SARS-CoV-2. The newborn was transferred to an isolation room under the NICU service for close monitoring. A pediatric ID specialist was consulted. Laboratory investigations were reassuring for age of the baby. The chest X-ray showed no significant abnormalities; the newborn remained asymptomatic and was discharged home after two consecutive PCR tests for SARS-CoV-2 resulted negative >24 hours apart. The newborn continued to remain asymptomatic and well upon follow-up.

Patient 4

An early-term, AGA male neonate, was born via uncomplicated scheduled C/S to a 23-year-old mother with a history of prior C/S and cholestasis in prior pregnancy. The baby was born vigorous and had normal APGAR scores after routine resuscitation at birth. The mother tested positive for SARS-CoV-2 using Cepheid rapid test two days prior to admission for scheduled C/S, and she remained asymptomatic throughout her hospital stay. Her laboratory investigations were within normal limits. After skin-to-skin care and colocating with the mother, the newborn was breastfed. He was tested following the baby’s first bath using OP/NP swab that resulted negative for SARS-CoV-2. However, a second PCR sent after 24 hours of life was reported as presumptive positive after the baby was discharged from the hospital. Following this, the newborn was seen in the pediatric ID clinic in addition to the general pediatrics clinic. His subsequent OP/NP swab PCR done on day 7 and 12 of life did not detect SARS-CoV-2. He remained asymptomatic and well upon follow-up.

Patient 5

An early-term, AGA female neonate, was born via uncomplicated C/S due to failed induction of labor of a 36-year-old mother with a history of diet-controlled GDM. The mother tested positive for SARS-CoV-2 using Cepheid rapid PCR test upon admission to the labor floor. Of note, the mother had symptoms concerning COVID-19 such as throat pain, chills, myalgia but no fever five weeks prior to the delivery. She was not tested for SARS-CoV-2 based on the testing criteria at that time. Her symptoms resolved in one week, and she remained asymptomatic in the following weeks, including her peri and post-partum course. The mother’s labs were significant for mild leukocytosis after delivery and elevated D-Dimer that was within range for the third trimester of pregnancy. The baby was born vigorous and had normal APGAR scores. After skin-to-skin care and colocating with the mother, the newborn was breastfed. The newborn was tested following the baby’s first bath using OP/NP swab that detected SARS-CoV-2. The newborn was transferred to an isolation room under the NICU service for close monitoring. Laboratory investigations were reassuring. The newborn remained asymptomatic during the hospital stay and was discharged after two subsequent tests for SARS-CoV-2 performed >24 hours apart resulted negative. She continued to remain asymptomatic and well upon follow-up.
